# Challenges and opportunities for healthcare workers in a rural district of Chad

**DOI:** 10.1186/s12913-017-2799-6

**Published:** 2018-01-08

**Authors:** Fabienne N. Jaeger, Mahamat Bechir, Moumini Harouna, Daugla D. Moto, Jürg Utzinger

**Affiliations:** 1 Swiss Tropical and Public Health Institute, P.O. Box, CH-4002 Basel, Switzerland; 2 University of Basel, Basel, Switzerland; 3 Centre Suisse de Recherches Scientifiques en Côte d’Ivoire, Abidjan, Côte d’Ivoire; 4 Centre de Support en Santé Internationale, N’Djamena, Chad

**Keywords:** Chad, Healthcare, Health services, Health worker, Nurse, In-depth interview, Motivation, Paediatric

## Abstract

**Background:**

Trained healthcare workers are an essential resource for effective health systems. However, healthcare workers’ perspective on healthcare, the challenges they face to provide quality health services, and opportunities to improve motivation and providing adequate care are rarely investigated in resource-constrained settings of sub-Saharan Africa.

**Methods:**

All reachable nurses of Abou Deia, a primarily rural district in the south-eastern part of Chad, were invited to participate. In-depth interviews were conducted to assess current challenges and opportunities faced in daily work, including factors that influence motivation and social wellbeing. Particular emphasis was placed on paediatric care.

**Results:**

Eight nurses were interviewed. Main work challenges pertained to overall workload, a lack of training and support regarding a serious case mix to be managed on their own, adverse working conditions, issues related to the local communities, and the impact of postings on nurses’ private life. Poor working conditions and perceived lack of recognition emerged as the main demotivating factors. Motivation to improve nurses’ skills so that they can provide good care, coupled with small, suggested changes in working conditions and health care organisation provide opportunities worth exploring to improve health workers’ satisfaction, motivation and the care they can provide.

**Conclusions:**

Health workers in a predominantly rural district in Chad face a wide variety of challenges, and hence their perspectives need to be taken into account to improve health services interventions that aim at enhancing quality of care. Nurses’ willingness to further develop skills and knowledge, proactive search of solutions to remedy stock-outs of drugs and other medical devices, and motivational factors to improve the quality of care represent important opportunities for improving health services for all.

## Background

Trained health workers are essential to any effective health system. Yet, critical shortages, inadequate skill mixes, and uneven geographical distribution of the health workforce pose formidable challenges in achieving universal healthcare coverage, particularly in low- and middle-income countries (LMICs) [1]. In Chad – a country which, despite petroleum exportations, remains very poor, ranking 186th on the human development index [2] – there are only three trained health workers (doctors, nurses, or midwives) per 10,000 population, while the World Health Organization (WHO)-defined minimum threshold considered necessary to ensure basic maternal and child health care is 23 health workers per 10,000 population [1]. This issue might explain, at least partially, why Chad is among the countries with the highest infant and under-five mortality rates; 72 and 133 per 1000, respectively [3]. Additionally, there is a very high maternal mortality rate; 860 per 100,000 live births [3]. According to a Multiple Indicator Cluster Survey (MICS) conducted in 2015, 34% of women in Chad delivered with a skilled birth attendant, of which only 22% took place in a health facility. The respective percentage is as low as 14% in rural areas [3]. Caesarean section rates were very low (1.5% of deliveries in 2010) [4]. In 2014, only 25% of children had received all the required vaccinations during their first year of life [5]. Chronic malnutrition concerns approximately 40% of children [5].

The Chadian health system further suffers from a notorious lack of funding with less than the agreed 15% of the national budget allocated to the Ministry of Health [5, 6]. According to the Chadian government, low oil-prices, increased expenses for security, and the poor international economic situation has contributed to this with the latter also reducing the influx of donor funds for health services [5]. The introduction of free care for pregnant women, children under-five, emergencies, and certain affections (e.g. HIV/AIDS and tuberculosis) in 2008, was not accompanied by adequate financial and human resources [6]. Hence, out-of-pocket payment remains a major source of financing for healthcare [6]. Insufficient drug supplies and frequent stock-outs further aggravate the situation and are at the root of a parallel drug market [5, 6]. Sanitary districts, comprised of a district hospital and multiple nurse-run health centres, are not always capable of providing even the minimal packages of care foreseen and some are completely non-functional [5, 6]. International organisations provide vertical programmes, while non-governmental organisations (NGOs) might provide health projects and sometimes even temporary hospitals, which are not always aligned and sustainable with the government’s strategic plans and resources [6].

Given the serious lack of medical doctors in many LMICs, there is a strong reliance on nurses to provide all kinds of curative and preventive care, especially in remote rural settings, and a tendency to shift tasks that principally should be performed by doctors to less qualified personnel [7, 8]. District hospitals in Chad are usually run by at least one medical doctor. Nurses of various training levels – depending on the degree (“Agent technique de santé” or “Infirmier diplômé d’état”), the training takes 2 to 3 years – run the health posts and health centres and work in the hospitals. Hence, nurses are supposed to deliver general out-patient care and are responsible for vaccinations and other outreach activities in their catchment area. Quite often they are supposed to perform deliveries on their own.

The health workforce is one of the key building blocks of any health system [9]. As such, the health workforce is considered an integral part of hardware essential to health services performance [10]. With the need to implement reforms and to improve healthcare delivery and quality of care, and in view of difficulties to retain health workers, especially in rural areas, understanding of health workers’ motivation and satisfaction has gained importance in health services research [11–17]. However, the front-line rural African health workers’ experience and the challenges they face on a daily basis are often ignored [18]. Understanding these challenges, in turn, is crucial to seize opportunities and activate health policy and planning. This issue is of particular importance in a country like Chad as there is a lack of health systems research, particularly with regard to health workforce’s experiences and satisfaction.

The purpose of this study was to investigate the health workers perspective and describe their sources of motivation, current challenges they face, and opportunities for improvement they see while delivering care in resource-constrained settings. This qualitative study was conducted in a primarily rural district in Chad, focusing on nurse-level healthcare providers.

## Methods

### Study setting and population interviewed

The study was carried out in Abou Deia, a district in Salamat region, in the south-eastern part of Chad. The district had an estimated population of 65,772 in 2008; thus, with a 2.5% natural yearly increase, it had approximately 72,600 inhabitants by the end of 2012, at the time the current study took place [19]. People heavily rely on crop farming, mainly mile for subsidiary purposes. In the study area, people may face periods with high acute child malnutrition rates. The main religion is Islam.

There is one district hospital, which is supported by the Centre de Support en Santé Internationale (CSSI), a local NGO, which has helped organise and finance additional, much needed staff such as a surgeon, an anaesthesia-nurse, and general nurses. The hospital has collaborated with Médecins sans Frontières (MSF), an international NGO, during the previous peak season of malnutrition until the month before the study started in October 2012. Additionally, there are seven health centres staffed with at least one nurse each.

All nurse-level (“Infirmier diplômé d’état” and “Agent technique de la santé”) healthcare staff of the entire district were invited to participate. This allowed coverage of nurses working at the district hospital and in the various health centres with different levels of equipment and accessibility, some very hard to reach, others close to main roads. Multiple attempts to reach nurses were undertaken for at least 2 weeks by mobile phone, local key informants, and face-to-face interactions to find a suitable date and time for an interview.

### Data collection and analysis

In-depth interviews with nurses were conducted at the location and time of their convenience; usually at their work place, after written informed consent was obtained. Particular attention was put on ensuring a private setting allowing the nurses to speak freely. Interviews were conducted in French – one of the official languages in Chad, which is very widely used by Chadian healthcare providers. An interview guide was used. The order of topics prompted was adapted according to the flow of the interview in order not to unnecessarily disturb the narrative. Nurses were encouraged to talk about their work and work experience, the difficulties they face on a day-to-day level in providing quality care, and how they organise themselves to overcome challenges, as well as motivational factors. Emphasis was placed on paediatric care, as child mortality remains very high, children represent a major patient group for in- and outpatient care, and reach-out policies and efforts are needed to improve care. Interview duration varied in length from about 30 min to more than 1 h, depending on the nurse interviewed. In general, nurses were eager to talk about their work experience, and hence, interviews usually lasted about an hour.

Interviews were recorded and transcribed. Transcripts were verified before analysis by listening to all the recordings, while reading the transcripts in parallel. Access to recordings and transcripts were restricted – no identifiable information would be made available to hierarchy and local authorities to ensure the possibility of free expression. Data were analysed using MAXQDA version 10 (VERBI Software Consult; Marburg, Germany), applying a thematic approach.

Furthermore, direct observation of the situation in the hospital and in parts in out-posts was possible as the study team stayed at the hospital and spent extra time observing services provided. Direct observation of work in health centres was possible in three health centres, though in one, it was a non-qualified aid providing care in the absence of the qualified nurse, while in another health centre, only the infrastructure could be visited. Three health centres were found with locked doors due to a nation-wide health services strike. The two medical doctors working in the district were also interviewed, although not being the focus of the current investigation, as the doctors’ status and work-situation is different. Additionally, a woman engaged by a local NGO to do community health promotion work with a main focus on malnutrition, vaccination, reproductive health, and HIV, was also interviewed.

The aforementioned nation-wide strike had been initiated by the “Union des syndicats du Chad” in response to the Chadian government’s withdrawal of the previously promised pay-rise for health services staff starting in 2012. The government justified the withdrawal with an increase in the military expenses to fight Boko Haram, lower petroleum prices, and the need for tighter budget control advocated by the International Monetary Fund. Commenced in July 2012, the strike movement persisted – despite strikes known to have a detrimental impact on population health [20, 21] – with a one-month break just before the current study was launched. At times, the strike committee even requested the refusal of minimal care leading to the complete closure of health infrastructures for several days. A deeper analysis of this strike and is effect on healthcare provision was not the purpose of the present study [22, 23].

## Results

Eight nursing-level healthcare workers out of 12 (66%) participated in the interviews: three women and five men; three nurses worked in the district hospital and five nurses in health centres. Of the remaining two health centres, one nurse could not be reached due to a national health services strike and one refused to be interviewed without giving any specific reason. Among the hospital nurses, two had also left the region due to the strike, both being employed by the government. Additionally, one untrained nurse aid volunteered for an interview in one of the centres, identifying himself as a nurse. His statements broadly matched the facility’s head nurse’s statements in the main domains except that the nurse aid, who claimed consulting himself, denied any needs for training or other major difficulties.

Several main areas of concern emerged, such as workload, tasks and duties, the work setting and working conditions, patient care and clinical capacities, poor recognition and support, the wish for more supervision and training, outside relations to patients and communities they serve, expectations from their superiors and policy, and the impact of work on nurses’ socioeconomic status and private life. Motivation to do a good job for the patients and the desire to improve skills were other prominent themes.

### Workload and quality of care

All three interviewed nurses working in the hospital described a mismatch between expected tasks and duties and available human resources. According to the doctor in charge, due to limited resources and frequent absences, staffing is organised to cover main programmed activities in the morning with a single nurse covering all nursing duties for the entire hospital during the remaining time as part of a 24-h work-shift. This is further aggravated by midwives only working day-shifts, and nurses additionally taking over their duties at night. All interviewed nurses expressed being overwhelmed and a sense of being left alone. *“A single nurse for an entire hospital, who also does the out-patient consultations, is a pharmacist, and midwife all in one. For all to be done, you are alone.”* All of the interviewed hospital nurses expressed concerns about the quality of care they provided under such conditions. *“You are alone. Sometimes you are obliged to not consider all parameters. You did not want to cause harm to the patient, you want to do good work, but you cannot anymore, you are overwhelmed”.*

A task shift towards nurses treating even severe cases (e.g. patients in shock or convulsing) all by themselves – increasing responsibility and burden on them – is reported with pride by some nurses. While hospital nurses can usually count on the doctor’s presence, health centre nurses report being mostly left all to themselves.

Receiving help to cope with the workload is further challenged by contextual factors as pointed out by the interviewees: the studied hospital is located far outside a small main town, which holds the advantage of being less called back to help out. Still, one nurse praised the times when new nurses all lived within the hospital compound and thus could help each other more easily. Furthermore, key personnel (e.g. laboratory technicians) are only present in the hospital during the morning hours; this, together with the distance to the small town, can cause serious delays in providing timely and adequate care (e.g. vital blood transfusions), as could be observed repeatedly during our stay. All interviewed hospital nurses expressed a sense of demotivation resulting from these contextual factors.

Health centre nurses expressed less difficulties coping with patient numbers, but they were more concerned with meeting the duties government policies foresaw. A nurse described the need for developing various strategies to be able to meet those different tasks, as means to fulfil them were not always given. The nurse described the population of the village he also lived in and their opinion as a source of motivation to seek solutions to overcome health services shortcomings and expressed wanting to meet their expectations:

*“Motivation is that you give yourself to work, you are proud of what you do with what you have available. People see it and you are proud. Don’t wait for the means to arrive; if there are no means they <the people> will say you are lazy.”*

Still, the influence of respected community members prioritising traditional medicine and in most case, for example, advocating against contraception, was mentioned as a challenge for nurses to comply with national policies.

### Work environment and general working conditions

Infrastructure and supplies may not only influence care for patients but also influence motivation, performance, and the wellbeing of staff. In the rural district hospital surveyed, poor planning at the time of construction and insufficient resources for maintenance had resulted in the hospital now only counting a few points, where water was available. Along with the notorious lack of soap and gloves reported, this hampers hygiene, which is essential for protecting staff and patients from nosocomial infections. Considerable distances within the hospital become a challenge for a single nurse when caring for multiple serious cases on different wards, a fact nurses would repeatedly complain about during observed shifts. The electricity generator only runs during surgeries, thus leaving the hospital in the dark most nights, and limiting treatment options to the main morning operation hours: *“After 13 hours there is a problem. We cannot switch on the generator – so the child will remain without oxygen-therapy despite signs of respiratory distress”.*

Hospital nurses were fully aware of the potential of, for example, oxygen-therapy and the fact that not being able to provide it may compromise patients’ outcomes. Two nurses in health centres even expressed relief about women choosing home delivery despite policies advocating for giving birth in facilities. Without running water and essential supplies, they feared deliveries. The availability of medical supplies and medication showed considerable heterogeneity among facilities. One nurse complained about a lack of rehydration salt, one about a lack of antibiotics, while a third nurse claimed to never face stock-outs at all.

Delayed arrival of essential supplies and stock-outs put pressure on nurses. Allocating limited stocks and potentially withholding treatment to save it for someone even more in need can be a challenging decision: during the study period, a health centre nurse hesitated giving the very last available antibiotics to a young adult with sever pneumonia. Limited medical knowledge – initially the nurse did not recognise the disease – combined with the need of taking resource allocation decisions at times of severely limited stocks, put considerable stress upon the nurse. Although observed only once, the nurse reported facing this occasionally.

The restriction imposed by the work environment and the lack of supplies to provide appropriate care were often accepted as given, but were perceived as disturbing. Still, some health centre nurses spontaneously reported organising medications proactively from various sources to remedy government supply shortages. Having to charge for such drugs, they say, sometimes leads to frustrations and accusations of malpractice by patients and their relatives.

Work environment factors can also directly influence the wellbeing, motivation and performance of health care staff.

*“Concerning motivation: the hospital is located far from town, you start at 8 a.m., you work, you have nothing to eat, you start feeling dizzy, you’ll give up on your patient and rest a bit, because there is nothing (to eat and drink), you won’t be able to stay up.”*

The two female nurses in the hospital identified similar minor low-cost changes as major potential motivation builders, asking they should urgently be addressed, such as a kettle to prepare tea, a cooling device for preserving food in the Chadian heat for their 24-h shifts, as there is no food available in the vicinity of the hospital, and no scheduled break during the long shifts.

### Clinical knowledge, referral practices, and hierarchy

Chadian nurses have to take medical decisions that influence patients’ outcomes. While hospital nurses state they can more easily seek the advice from a doctor, health centre nurses are often isolated, and hence, they have to decide on their own, despite potentially only having limited clinical experience. When a patient presents with a severe or unclear condition, the health centre nurse is required to refer the patient to the district hospital for a second opinion or advanced care. Two health centre nurses admitted often trying to treat even severe cases themselves. Reasons given were (i) geographic isolation, (ii) referrals being perceived as cumbersome and often refused by patients, and (iii) the misconception that, as long as there is no operation, no difference in care between hospital and health centres can exist. One of the health centre nurses expressed the wish to be able to call a physician for medical advice more easily, while others accepted the isolation as given. Poor mobile phone network coverage at the time of the survey, a need to pay for such calls out of their own pocket, and the current practice to only call to organise evacuation of severe cases, combined with poor support and knowledge gaps, are reasons mentioned by local doctors and nurses on why nurses do not seek expert advice and risk to potentially install suboptimal care instead.

All but one junior nurse – independent on whether they worked at a hospital or a health centre – expressed the need for improving their clinical decision capacity and knowledge-base, with female nurses more easily expressing knowledge gaps in every day work: trainings, protocols, and a library for the hospital were mentioned as critical support. *“A library - that would help a lot, with essential books and everyone can come in the afternoons and learn – we don’t even have that here, we are completely abandoned”*. Being sent for training was perceived as an incentive, as a way to improve care provided, and to increase the likelihood for a next posting being in a better facility and a more attractive location.

According to the nurses, supervision by the district doctor is mainly done through a review of patient numbers and activities reported. Two health centre nurses expressed their wish for a more hands on supervision by a medical doctor who would practice with them for a day, to whom they could present complicated cases, and from whom they could learn. *“We need further training; formative supervision is missing, we would need a doctor spending the day with us, see what works and what not.”*

During the time spent with nurses, the interviewer observed several medical shortcomings, such as the administration of adult dosages of paracetamol to infants, which can be harmful, but are hard to detect without direct observation. Discussing such issues with concerned nurses was well received, which underscores that nurses are keen to improve their knowledge and skills. Nurses in the hospital are helped in parts by the presence of a doctor and by morning staff assembly which is used for teaching: *“Morning assembly helps us a lot. You see the patient, you present, everyone discusses the case.”* There was a common desire for improved teaching, training, and support in decision making, be it for personal or career development reasons.

### Local community

Being accepted by the community was of utmost importance to nurses living in the communities and was regularly mentioned as a source of motivation. The desire to comply led to difficulties and sometimes suboptimal care. A hospital nurse mentioned the need to rely on daily injections for hospitalised patients, as the population would not believe in the effect of oral medication. A health centre nurse reported:

*“They just want injections. If you do well your treatment, you still need to do a bit of placebo (normal saline or dexamethasone) injection. Even for something that does not need an injection we do it. They won’t swallow the tablets, but once you put the needle, there you treat, there they understand. <…> Otherwise they won’t come back.”*

The nurse claimed the district medical doctor had suggested this procedure to meet patient expectations and to increase compliance with oral treatment. The nurse also reported that initial refusal of a young colleague to do so, had led to patients flocking to another health centre until he also started the medically unnecessary injections. Although knowing that such practices reinforces misconceptions among the communities, nurses did not feel equipped to do the necessary outreach and information work themselves.

Arrival of new NGO healthcare programmes and projects can be challenging for health workers, as expectations may rise in the community on provided care and goods that cannot be kept-up once the NGO leaves. The area had seen much appreciated help by MSF during the latest food crises, only weeks before the interviews. Nurses in several health centres and the hospital reported still using MSF-leftover medical stocks to cope with government supply shortages. MSF had handed out free insecticide-treated nets and free soap to all those who attended; a service government facilities cannot keep up with.

*“The mothers want things like bed-nets and soap. MSF gives a lot of things. Now that MSF has left, people enquire, news spreads in the villages, and they know that those people (MSF) have left. The people do not come for consultations anymore. Before MSF it was ok, but now there is nearly zero (attendance).”*

Hospital nurses felt that now that these items were no longer available for free distribution, there was a drop of attendance even below pre-MSF rates. They disliked that some members of the population seemed to blame them of misconduct when not providing items free of charge any longer. The same was reported for medication sold when the free-government supplies for paediatric patients were finished. Two nurses urgently asked for insecticide-treated nets for pregnant women and ready-to-use therapeutic foods – the latter sometimes miss-used as sweet treats instead of treatment – to motivate consultations.

In general, nurses also expressed a certain difficulty regarding patient care due to differences in backgrounds: *“We came from far away; we do not know local customs; all that is very difficult.”*

Five nurses expressed major concerns regarding health concepts, traditional medicine, and late presentation of patients. In particular, children arriving in poor conditions was sometimes identified as being frustrating and considered entirely the parents fault by nurses during hospital cases observed. At times, this even resulted in a reproachful attitude of the nurse towards parents.

### Socioeconomic situation and family expectations

#### Salary and family expectations

Although not a predominantly emerging theme, some nurses did mention insufficient salaries being a challenge and a demotivation factor. A hospital nurse employed by a local NGO regretted that the salary did not allow saving money, thus impeding sub-specialty training and career development. As the position of a nurse is perceived as in a financially secured position, extended family members expect support. *“They say: why do you work, you have a salary... What salary, it’s not even enough for half a month!”* Two nurses suggested therefore that they would like to receive “motivation” money. One government-employed nurse mentioned additional farming activities to increase his income. Reports of a staff stealing medication to brush up his salary were also given.

Still, there were also reports of being grateful for having a job. None of the female nurses reported the small salary as a source of demotivation. One mentioned spontaneously she would have been ready to accept even a slightly lower government salary if it allowed her to do government-working hours, which she perceived as better.

#### Posting and social life

Posting may have a considerable impact on the nurses’ private lives and the care they can provide. Staffs’ influence – especially in the case of junior nurses – on where they get posted is very limited. While moving to pursue work in remote locations was generally accepted, it did not always come easy on nurses. One female nurse expressed difficulties about having been posted in a remote village with her son, her husband being posted in the capital, more than 700 km away. With public transport hard to reach and unstable mobile phone networks, this likely puts a strain on the young family. She complained about the difficulties regarding finding healthy, varied food in the absence of local markets at her remote posting. According to one of the physicians working in the district, security issues may also arise for unaccompanied female health workers in the periphery.

Another freshly graduated nurse who was posted in a small remote village expressed difficulties about being accepted by the local community and feeling lonely. The nurse identified young age, and different ethnic and language background as main reasons for challenges in integration and acceptance. According to a nurse in a neighbouring health post, his initial attempt to follow medical standards despite the preference of patients for injections may have further hampered integration. With the health post and the nurse’s quarters at the outskirt of the village, the nurse pointed at the poor living conditions and made our research team visit his room that was equipped with a mattress, a cooker, cardboard boxes for storage, and a machete for defence, as there were frequent encounters with hyenas at night.

### Motivation, demotivation, and line management

When asked about key motivation to pursue their profession, two nurses expressed the desire to help sick people; one of them had lost several siblings due to lack of access to quality care. Inappropriate working conditions and lack of recognition and sometimes even the feeling of unjust judgement by senior staff were the main factors hampering motivation. *“Some remarks and the lack of recognition for our work done is demotivating – and that when we do mistakes, the difficult conditions are not taken into account”*. Lack of recognition by patient families and health services hierarchy, and lack of encouragement, which can also be financial, were the predominant demotivation factors in all hospital nurses who suffered from extensive work load and long shifts without adequate recovery, lack of basic commodities during shifts (lack of food storage), and the feeling of being held responsible for sub-optimal care due to difficult conditions by patients and sometimes even health services’ hierarchy. Lack of material to provide care, but also factors related to their posting in remote rural areas (e.g. lack of markets and as such the difficulty to buy fruit or meat), were factors of demotivation in the periphery.

Supervision and training dominated as factors with the potential to increase work motivation, followed by setting-specific factors. Three health centre nurses asked for supplies, saying that this can be used to motivate villagers to come for vaccination, helping them to improve the vaccine rates they have to report. They mentioned ready-to-use therapeutic foods that are appreciated by the population wrongly as sweet treats but actually should only be given out clearly branded as a treatment for malnourished children for re-feeding policies to work, and insecticide-treated nets. One government health centre nurse also asked for a health promotion outreach specialist to address prevention issues in the served communities. The interviewed female health promoter working in the district confirmed that due to limited resources and logistic difficulties, only a limited number could currently be reached.

#### The health services strike

All participating hospital nurses were employed by a local NGO and two health centre nurses by UNICEF and all of them kept working despite the strike. Three health centre nurses were government employed and enforced the strike – which aimed at increased nursing salaries -- to various degrees, including a complete shutdown of a facility even for emergencies, as the assigned nurse had left the location. Salary issues and allowances for motivation purposes were mentioned by one government and one NGO employed nurse when prompted about motivation.

A certain pressure by government nurses on non-government-employed nurses to participate in the strike was reported of the record by hospital nurses. One female health centre nurse explained that for her it would be impossible to follow a strike, while living in the community she serves.

## Discussion

Adequate human resources and good performance of healthcare workers are essential for any effective health system [9]. Multiple factors can either enable or disable nurses to provide optimal care. This study highlights several challenges as perceived by nurses in rural Chad. Our research also shows potential avenues to increase work satisfaction and to improve patient care, particularly through improved working conditions and case-solving capacities.

### The front-line health workers’ perspective

The main challenges potentially impacting patient care, motivation, and work satisfaction that were highlighted by the interviewed nurses were related to a mismatch between expected tasks and duties and available resources in terms of staffing, essential infrastructure, and supplies. This was aggravated by a work environment and working conditions disregarding nurses’ personal needs both in term of organisational (e.g. long working hours) and structural nature (e.g. lack of basic commodities at work) and social life. Concerns about poor patient care, clinical knowledge gaps, insufficient support and clinical-decision making aids, and a perceived lack of recognition put strains on nurses with the risk for demotivation, drop out, and burn out [14, 24]. The situation was further aggravated by remote postings far away from family and friends.

Identified challenges, such as heavy workload, limited clinical case management capacities, insufficient training and decision aids, inadequate supervision, low salaries, poor recognition, and frustration about not being enabled to perform according to common professional standards, have been identified as key motivation or demotivation factors for health workers and health services performance [12–16, 25–27]. Still, it is rare to identify so many aspects demonstrated in a single study and to find such a close up on the nurses’ perspective, especially concerning the impact on their lives and wellbeing related to work. Concerns regarding basic commodities at work regarding staff’s wellbeing, insufficient knowledge and needs for changed support, and referral practices and outreach, as well as impacts on private life, were additionally revealed by our research.

### Gender-specific issues

It is worth pointing out that, despite not the primary focus of our investigation, several gender-related issues emerged. Although there might be a potential bias due to the small sample size, issues identified may warrant further scientific inquiry, as they are rarely studied in relation to health workers and may need to be accounted for, if females are to be taking on a more important role in healthcare. Female health workers in Chad seemed more concerned with basic commodities such as alimentation during work, potentially because they are traditionally the ones catering for themselves, males and their families. Fastone et al. have also demonstrated female health workers being more concerned with living conditions and schooling opportunities [28]. Monetary aspects seemed less relevant for female respondents and postings in peripheral health centres rarely encountered with a single female nurse working in a health centre in Abou Deia district. Similar findings have been reported from Burkina Faso [17] that might be explained by traditional society values and barriers, and potentially even security issues. Females being more likely to admit knowledge gaps and errors may have been influenced by the interviewer being female. Research points at females being more distressed by critical care [29], a finding potentially also holding true in the Chadian setting studied here.

### Salaries and allowances

Interestingly, inadequate salaries and lack of incentives, dominant demotivation factors in other studies [15, 30–33], seemed less of a priority concern to Chadian nurses, with only two nurses expressing financial difficulties and the desire for adequate financial recognition of their work, spontaneously, but all of them stressing concerns about quality of patient care with available means and their working conditions. This finding may have been influenced by a selection bias imposed by the fact, that due to the national health services strike for higher salaries, those nurses most concerned with remuneration had left the area, but also by other issues being more challenging in a country where having an employment is already considered of foremost importance.

### Working conditions and staff wellbeing

Excessive workload, understaffing, and time constraints [30, 34], have been identified as major stress factors for nurses in other African settings [35, 36]. Stress related to work is a frequent but not the only potential health hazard in health care [35, 37]. The fear of contracting infections – a health hazard that is a major worry in health workers in South Africa, a country with high rates of HIV and awareness of related health risks for employees [35, 38] – was not spontaneously mentioned in Chad, despite inappropriate hygiene conditions in all facilities visited. Although the lack of water, soap, and gloves was mentioned, improvement of these conditions and rising of awareness of nosocomial infection is warranted.

Besides the lack of supplies, equipment, and infrastructure [14, 25], basic work environment factors, working conditions, and commodities regarding staff wellbeing (e.g. enabling safe food and water storage and access during long shifts in extreme climates), have received little attention in research, probably as it seems too trivial or because the conditions are considered normal. Yet, our research reveals that these issues are a predominant concern, especially to females who have to cater for themselves. Interviews with the hospital nurses indicated that simple commodities, like the possibility to keep food in a fridge or boil water for a tea, or scheduled resting times, were considered highly motivating. It may be worthwhile to consider such aspects also in other resource-constrained or climate exposed settings, thus trying to improve working conditions to motivate for good clinical care and increase the attraction of health professions to reduce the workforce shortage in healthcare. It is clear that without appropriate infrastructure, material, and supplies, and processes and work-organization adapted to patient and staff needs, performance is hampered.

### Clinical knowledge, referral practices, and hierarchy

Teaching and training and resulting career development opportunities are motivational factors in Chad and elsewhere [12, 14, 39, 40] and, together with supervision and mentoring, hold promise to improve standards of care. Current supervision focuses on checking government indicators and a review of clinic registers. Supervision, as desired by nurses, that allows for exchange and hands-on training holds promise to identify mistakes, empower nurses in their decision making, and foster trustful relations with line managers. It may help appreciate nurses’ work under often difficult circumstances but also allows detecting inadequate performance of certain staff. Lack of appreciation and recognition [12, 13, 26], and thus an unsatisfying working atmosphere, was a frequently mentioned concern also in Chad, indicating the need to improve line-management and respect for lower staff-grades. Recognition, management issues [14], and satisfaction with supervision [33] are core factors also to staff retention. Salaries may be considered a form of recognition and the government’s withdrawal of a promised salary rise a lack hereof. Considering that in Kenya, support of the Ministry of Health had been identified as a major factor influencing satisfaction [27], finding ways to increase health funding and management in Chad is urgent.

In the light of improved mobile phone networks and new technologies, expressed uncertainties and knowledge gaps regarding optimal care for patients, clinical case management support, decision-aids, and referral practices need to be re-evaluated. A common internal communication network free of charge to the local health workforce, coupled with a change of policy promoting to call more qualified healthcare workers for advice in case of insecurity, and an improved work atmosphere and awareness of competences may help improve quality of care and actually reduce costs and harm linked to unnecessary referrals, loss to follow-up, or inadequate treatments. It may reduce stress and the feeling of being left alone, particularly among nurses in remote settings, and promote professional growth.

### Implications for health services

When trying to improve health services and implementing reforms, the main focus is on improving care for patients. Efforts to improve quality of care have been shown to improve motivation [16]. While this needs to remain the primary focus, health services should not only be evaluated from a patient, but also from a health worker’s perspective. Doing so will help to insure that healthcare workers are enabled to perform well.

The Chadian nurses expressed motivation to care well for their patients, to fulfil community expectations and policy requirements, and a desire to improve skills, knowledge, and career chances making them open for training and supervision, which in turn might improve quality of care. At times, nurses proactively searched for solutions to address healthcare problems (such as in case of stock-outs), which implies willingness to change and perform well. This represents an opportunity for health authorities when launching improvement projects. The nurses knowledge of the local communities, needs, and health services may help identify setting-specific solutions. Nurses mostly wish to be accepted by the host community [31]. Willing to work in different settings, greater care should be placed on matching nurses with communities regarding language and cultural acceptance to help reduce stress among healthcare workers and improve external relations and mutual understanding. This may be further improved by coordinated health promotion activities, as suggested by study participants. There is a need to sensitise traditional leaders and communities regarding health services and basic concepts of modern medicine and information on when to attend, as well as by the reduction of stock-outs of free supplies, during which – not only in Chad – front-line workers sometimes feel wrongly accused by communities [41]. Some nurses may also require support in dealing with frustrations, as reproachful attitudes towards patients’ family members may lead to a decrease in attendance.

### Limitations

Only eight out of 12 invited nurses participated in the current study. Despite small numbers, a surprisingly substantial and extensive panoply of issues relevant to nurses emerged. The additional emphasis on challenges and opportunities next to motivation, as well as on concrete examples derived from paediatric care allowed the nurses to share more extensively also on issues that may not be considered determining for motivation and satisfaction, potentially accepted as given, but still relevant to patient care. The qualitative approach combined with time spent with direct observation and building trust allowed a close-up on the nurses’ perspective and hopefully a reduced impact of social desirability. The approach also allowed identifying easily addressing commodity issues such as basic alimentary difficulties mentioned by female nurses, knowledge gaps, and the need for a new referral system, which are not usually identified in research focusing on nurses. Next to health services aspects, our research strategy also allowed to expand to community relations, demonstrating the communities as a source of motivation but also the need for further out-reach health promotion activities. Despite small numbers a good degree of saturation could be achieved with nurses confirming a consistent picture of the overall situation in this health district of Chad with additional interviews no longer adding new aspects to be explored. Furthermore, expressed views and concerns could, at least partially, be confirmed by direct observation and conversations with several other levels of health care staff, not presented here.

Small numbers and difficulties to recruit government-employed nurses may have induced bias, especially concerning patient and community commitment and salaries and may have aggravated the workload for hospital nurses. Working for an NGO, fear of retaliation, moral dilemmas, and the wish not too loose acceptance in the host communities may have influenced the degree to which the strike was enforced and why many government-employed nurses had left the region. Still, except for a slightly lower salary, challenges articulated were the same for government and non-government nurses, as their tasks are the same.

Our findings are also relevant for other resource-poor settings, particularly those where provision of care and management of health services are similarly challenging due to difficult access (such as, where large distances, flooding during the rainy seasons, and poor or even inexistent roads renders support, supervision, and referrals difficult), excessive heat (e.g. challenge for food and drug storage), a very limited workforce (nurses often working alone in very remote settings), or security issues – factors shared in many areas of the Sahel. Issues raised regarding communities and postings of staff may be particularly relevant in other settings with multiple cultures and languages.

## Conclusion

Nurses in a primarily rural district of Chad face many challenges in their day-to-day activities. Generally committed to patient care, they emphasised a desire for recognition of their efforts, and improved working conditions, support through supervision, teaching, and training. Next to health services factors such as workload, poor infrastructure and supplies undermining provision of care, contextual factors related to their postings and living conditions were among the challenges identified. There is a pressing need to address these issues to enhance nurses’ motivation and ability to perform.

Investigating challenges faced by nurses by means of in-depth-interviews of a small sample, allows identifying basic needs that warrant addressing. Reports from Chadian nurses on how they address these challenges successfully and their proposed small-step solutions should be evaluated and considered as a learning opportunity for change from within.

Nurses are an essential health service resource but the human aspects should not be neglected. Taking on not only a political, health service, or patient related perspective, but also the front-line health workers’ perspective is important to foster sustainable, well-functioning health services. If health workers shall perform well, they need to be accountable for their work [25] but also enabled to perform according to best practices while maintaining their own health and wellbeing. Basically, human beings, patients, and staff need to be in the centre of attention for health services to be successful.

## Abbreviations

CSSI: Centre de Support en Santé Internationale
LMICs: Low- and middle-income countries
MSF: Médecins sans Frontières
NGO: Non-governmental organisation
UNICEF: United Nations Emergency Children’s Fund
WHO: World Health Organization
